# Semilobar holoprosencephaly with cebocephaly associated with maternal early onset preeclampsia: a case report

**DOI:** 10.1186/s13256-018-1647-6

**Published:** 2018-07-07

**Authors:** Ahmed Amdihun Essa, Lakachew Asrade Feleke, Dawed Muhammed Ahmed

**Affiliations:** Department of Obstetrics and Gynecology, Bahir Dar University, College of Medicine and Health Sciences, Bahir Dar, Ethiopia

**Keywords:** Cebocephaly, Micropenis, Polydactyl, Preeclampsia, Case report

## Abstract

**Background:**

The term holoprosencephaly was proposed by DeMyer and Zeman. It is a developmental defect of the embryonic forebrain with heterogeneous etiology including genetic and environmental factors. It is commonly associated with midfacial defects and has a spectrum of presentations. There are four types: alobar, semilobar, lobar, and variant. Holoprosencephaly is relatively rare. The overall prevalence in a multicenter study was 1 in 13,000 to 18,000 live births. However, the presentation of holoprosencephaly with cebocephaly, micropenis, agenesis of middle phalanges of the fifth finger, and postaxial polydactyly in association with early onset preeclampsia is extremely rare. We report a case with a constellation of the above congenital anomalies.

**Case presentation:**

A 34-year-old gravida II para l woman presented to Felege Hiwot Referral Hospital with the diagnosis of semilobar holoprosencephaly and early onset preeclampsia with severity features. The gestational age at admission was 26 + 3 weeks. She is Amhara by ethnicity. The pregnancy was from a non-consanguineous marriage. She presented with the complaints of severe and persistent headache associated with blurring of vision and generalized body swelling. After she was stabilized, she and her husband were counselled and termination was decided. She gave birth after three doses of 100 microgram misoprostol given vaginally every 3 hours. The outcome was 1.1 kg male neonate; there were associated dysmorphic features of holoprosencephaly such as cebocephaly, micropenis, and postaxial polydactyl with agenesis of middle phalanges of the fifth finger. Only basic care was given and the neonate died after 20 minutes’ stay in our neonatal intensive care unit. The mother was counselled to have preconception and antenatal screening in her next pregnancy. She left the hospital relatively well.

**Conclusion:**

In women with a history of holoprosencephaly or holoprosencephaly in the current pregnancy, antenatal workups should include workup for fetal chromosomal disorders and metabolic workup for maternal preeclampsia. Sonographic diagnoses of holoprosencephaly always need a careful search for other congenital anomalies. In the severe forms, early termination should be counseled for its poor prognosis. Associated severe congenital anomalies and severe morbidities of the survivor can be discussed while counselling.

## Background

The term holoprosencephaly (HPE) was proposed by DeMyer and Zeman. It is a developmental disorder resulting from failure of septation, cleavage, or differentiation of the midline forebrain structures at various levels or to various degrees which result in a defect of the embryonic forebrain. It is commonly associated with midfacial defects and has a spectrum of presentations. There are four types: lobar (presence of an interhemispheric fissure but the cingulate gyrus and the lateral ventricles are fused, and there is no septum pellucidum); semilobar (posterior partial formation of the interhemispheric fissure, with only a single ventricle); variant (heterotopic gray matter); and alobar (absence of the interhemispheric fissure, falx cerebri, the third ventricle, and fused thalami, and often absence of neurohypophysis and olfactory tracts) [1]. HPE is relatively rare. Worldwide prevalence is difficult to estimate. The overall prevalence in a multicenter study was 1 in 13,000 to 18,000 live births with female preponderance [2]. However, the presentation with cebocephaly and association with early onset preeclampsia is extremely rare in the literature.

## Case presentation

We describe the case of a 34-year-old gravida II para l woman, with a gestational age of 26 + 3 weeks at admission, who had a relatively healthy 4-year-old child with her 40-year-old husband of non-consanguineous marriage. She had been on injectable contraception for 2 years and had regular menses for 6 months before the pregnancy. She had antenatal care at a local health center and was vaccinated with tetanus toxoid once and supplemented with iron for 3 months. She was screened for retroviral infection, hepatitis, and syphilis and it was documented nonreactive. She had no anatomic scan at early gestation. She came to Felege Hiwot Referral Hospital with the chief complaint of severe and persistent headache of a day’s duration which was occipital in location associated with blurred vision and generalized body swelling of 1 week’s duration. She had no other danger signs in pregnancy. Her past gynecologic history, medical history, and surgical history were uneventful. She is Amhara by ethnicity. She had no known family history of hereditary or chromosomal disorders.

Her blood pressure at admission was 180/120 mmHg and pulse rate was 84 beats per minute; her respiratory rate was 22 breaths per minute and she was afebrile. She had pink conjunctiva and non icteric sclera, 24 weeks-sized gravid uterus, no abdominal tenderness, no organomegaly, no sign of fluid collection in her abdomen, and the fetal heart beat was positive. She had no vaginal bleeding or discharge. She had pedal and pretibial edema. She was conscious and oriented to person, place, and time. Her deep tendon reflex was +2 and her motor and sensory examinations showed no motor or sensory problems. Other parts of systemic examinations were normal.

**Fig. 1:**
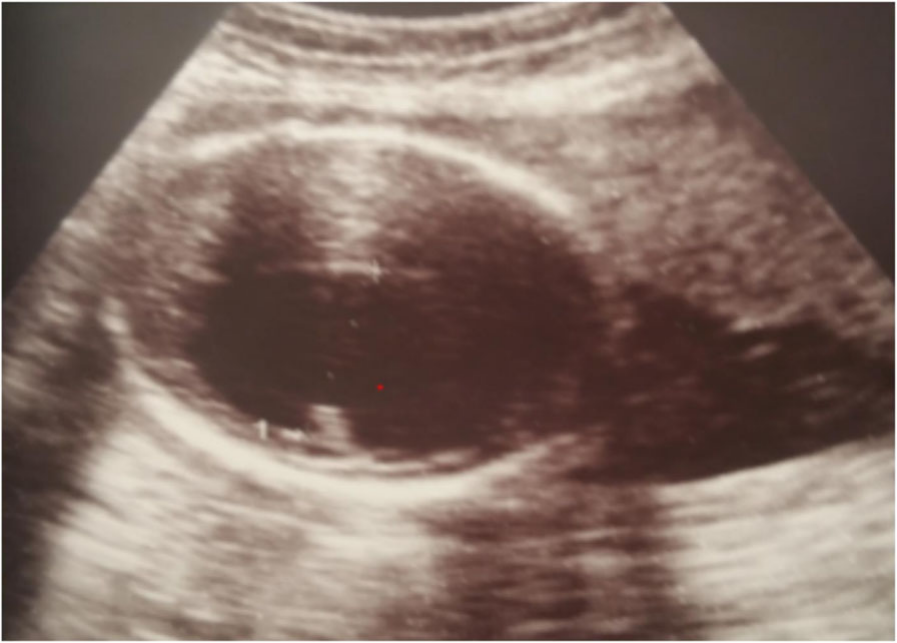
Ultrasound image shows axial view of semilobar holoprosencephaly (see partially formed falx cerebri and absent cavum septi pellucidi)

Her hypertension was controlled with intravenously administered hydralazine 5 mg two doses at our emergency department. In her complete blood count her white blood cells were 7300 cells/micL, hemoglobin of 13.4 g/dl, and platelet count was 169,000 cells/micL. Urine protein dipstick was +2, and liver and renal function tests were done: serum glutamic pyruvic transaminase (SGPT) 89 IU/L (elevated), serum glutamic oxaloacetic transaminase (SGOT) 102 IU/L (elevated), alkaline phosphatase (ALP) 229 IU/L, and lactate dehydrogenase (LDH) 288 IU/L. Total bilirubin was 0.24 mg/dl, albumin was 3.49 g/dl, blood urea and nitrogen was 12 mg/dl, serum creatinine was 0.69 mg/dl, and oral glucose tolerance test was in the normal range. Obstetric ultrasound showed a singleton, alive, intrauterine pregnancy with average gestational age of 26 weeks, there was a single large ventricle with partially formed midline structure (see Fig. 1), amniotic fluid index was 13.4 cm, placenta was located anteriorly at the body of the uterus, and the presentation was breech; the fetus had normal four chambers of heart with normal outflow tract.

**Fig. 2:**
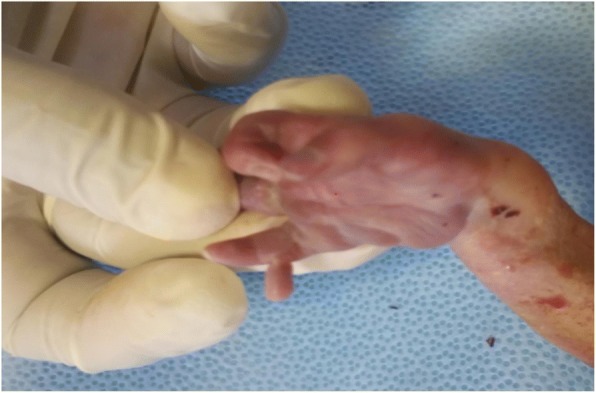
Postaxial polydactyly with agenesis of middle phalanges of the fifth finger

**Fig. 3:**
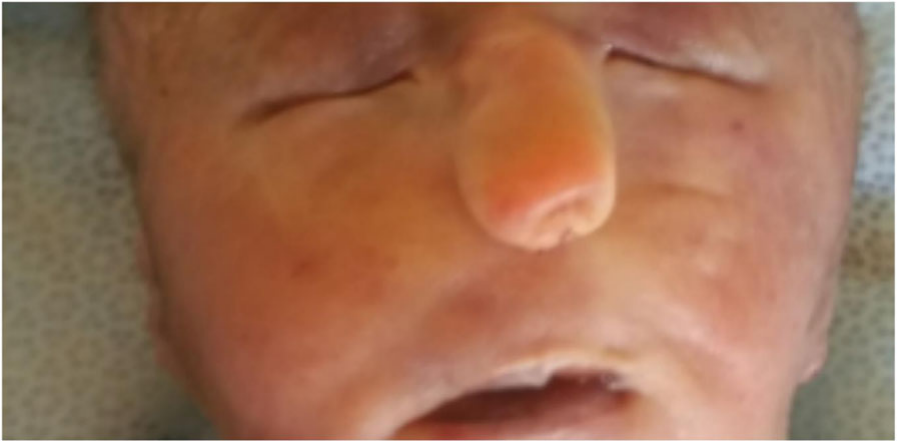
Cebocephaly (proboscis-like nose with single nostril and absent philtrum and moderate hypotelorism)

**Fig. 4:**
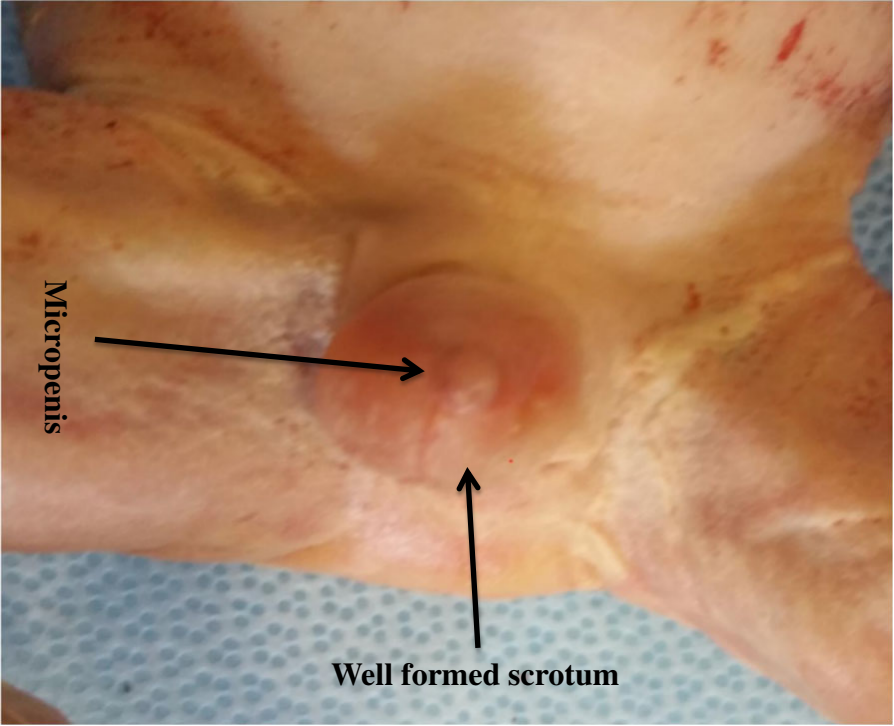
Micropenis of 8 mm length with well-formed scrotum

After blood pressure was controlled (it took 2 hours), she was admitted with the diagnosis of late second trimester pregnancy and preeclampsia with severity feature plus semilobar HPE. Seizure prophylaxis for preeclampsia was given (magnesium sulfate according to World Health Organization guideline), methyldopa 500 mg orally every 8 hours was added, and she was counselled about options of management; the high incidence of associated anomalies, severe morbidities of survivors, and poor prognosis were discussed. Termination was decided and done with misoprostol 100 microgram every 3 hours at the third dose with outcome of 1.1 kg male, alive neonate. On examination of the neonate, there was cebocephaly, hypotelorism, single patent nostril which enabled nasogastric tube 6F, micropenis (8 mm), and unilateral right hand polydactyly with agenesis of middle phalanges of the fifth finger. There was rigidity involving all extremities which resisted extension and flexion (see Figs. 2, 3 and 4).

After basic neonatal care was given (cord tied, airway cleaned, and newborn dried), he was transferred to our neonatal intensive care unit (NICU) but he died 20 minutes after admission to NICU. Immediate cause of death was not known. Following his death, further investigations were not possible for cultural reasons. At third postpartum day, maternal blood pressure was 130/90 mmHg, pulse rate was 78 beats per minute, and respiratory rate was 20 breaths per minute. Her complete blood count showed white blood cells of 12,000 cells/micL, hemoglobin was 11 g/dl, and platelet count was 122,000 cells/micL. Liver function tests showed SGPT of 35 IU/L, SGOT of 12 IU/L, ALP of 359 IU/L, and LDH of 254 IU/L; total bilirubin was 0.56 mg/dl, blood urea and nitrogen was 22 mg/dl, and serum creatinine was 0.8 mg/dl. After she was counselled to have preconception care and prenatal screening in next pregnancy, she was sent home relatively healthy. She was well at postpartum visits and methyldopa was discontinued at seventh postpartum day.

## Discussion

HPE is the most common congenital malformation of the developing forebrain and midface in humans. The etiologies of HPE are heterogeneous, including genetic and environmental factors. Almost half of all cases have cytogenetic abnormalities and as many as 25% have a documented monogenic syndrome [3]. Multiple environmental factors have also been reported in the pathogenesis of HPE, including: maternal diabetes, a 200-fold increase in the incidence of HPE in babies of diabetic mothers over babies of non-diabetic mothers was reported; radiation or toxin exposure during pregnancy; toxoplasmosis, other (syphilis, varicella-zoster, parvovirus B19), rubella, cytomegalovirus, and herpes (TORCH) infection; cigarette smoking; and retinoic acid [4, 5].

It is commonly associated with anomalies such as hydrocephalus, agenesis of the corpus callosum, posterior fossa abnormality, cerebellar vermis aplasia, myelomeningocele, absence of an olfactory bulb, a cleft lip/palate, adrenal hypoplasia, renal dysplasia, renal cysts, omphalocele, cardiovascular malformations, intestinal abnormalities, club foot, sirenomelia, microcephaly, postaxial polydactyly [6], spina bifida, and endocrinopathies such as pituitary gland dysplasia, growth hormone deficiency, and diabetes insipidus [7].

In this case report, karyotyping was not done but maternal age was 34 years and the fact that she developed severe preeclampsia remote from term and the constellation of neonatal anomalies can suggest a possible associated chromosomal disorder; most likely trisomy 13. Phenotypically, HPE varies widely. The classic saying of “The face predicts the brain” is correct in approximately 70–80% of cases for pediatricians involved in their management [1]. The reverse “The brain predicts the rest” may be the rule for obstetricians who follow a fetus with HPE antenatally to look for associated anomalies in every system which may have prognosis value. In this case report the facial, musculoskeletal, and genital anomalies were missed at sonographic evaluation.

The HPE facies which are characterized by hypotelorism, are grouped into five major categories: (1) cyclopia, a single eye or partially divided eyes in a single orbit with a proboscis above the eye; (2) ethmocephaly, severe hypotelorism and a proboscis between the eyes; (3) cebocephaly, hypotelorism with a single nostril and a blind-ended nose; (4) absent intermaxillary segment with central defect and hypotelorism; (5) intermaxillary rudiment with hypotelorism [8]. The unusual feature in this case report is an interorbital proboscis-like nose with patent single nostril (which enabled passage of nasogastric tube) with intact lip and palate, which could be a variant form of cebocephaly associated with semilobar HPE. It is very difficult to distinguish from ethmocephaly but the presence of moderate hypotelorism, the location of proboscis at lower than eye level, patent single nostril, and absent philtrum fit with cebocephaly. An autopsy examination would have been very helpful.

Furthermore, HPE was reported in association with diabetic mellitus in few case reports. One case report mentioned a newborn with alobar HPE associated with cebocephaly and micropenis in a Klinefelter fetus of a mother with type 2 diabetes mellitus with obesity and poor metabolic control [9]. There is also literature which reported an association between trisomy 13 and preeclampsia [10] but this case report showed a specific association of HPE with early onset severe preeclampsia which can have risk of morbidities and mortality to the mother.

The survival of a newborn with HPE depends on the severity and associated anomalies. Most children with alobar HPE have a poor prognosis. Children with other forms of HPE rarely survive into adulthood. HPE survivors can have the inability to smell, developmental delay, profound intellectual impairment, and seizures. Other problems include: (1) increased muscle tone to the point of spasticity and contractures; (2) fluctuating behavior between calmness and irritability; (3) voice abnormalities; (4) difficulty with swallowing; (5) growth delays; (6) sleep disturbances; (7) periodic brain stem and/or hypothalamus dysfunction with irregular breathing, heart rhythm, heart rate, and unstable temperature control; and (8) pituitary and/or thyroid gland dysfunction. These are also the common causes of death in HPE [11].

### Differential diagnosis

- Smith–Lemli–Opitz syndrome
- HPE-polydactyly syndrome

## Conclusions

In women with a history of HPE or HPE in the current pregnancy, the antenatal workup should include a genetic workup to rule out fetal chromosomal disorders and metabolic workup to rule out diabetic mellitus. If a woman decided on expectant management for fetus with HPE, maternal screening for preeclampsia should be added. Sonographic diagnoses of HPE always need a careful search for other anomalies. In the severe forms, early diagnosis and termination should be counseled for its poor prognosis. The severity of HPE, associated neonatal anomalies, possible severe morbidities of survivors, and possible maternal risks such as preeclampsia can be used for counselling.

## Abbreviations

ALP: Alkaline phosphatase
HPE: Holoprosencephaly
LDH: Lactate dehydrogenase
NICU: Neonatal intensive care unit
SGOT: Serum glutamic oxaloacetic transaminase
SGPT: Serum glutamic pyruvic transaminase
